# Confirmation of a Phenotypic Entity for *TSPEAR* Variants in Egyptian Ectodermal Dysplasia Patients and Role of Ethnicity

**DOI:** 10.3390/genes13061056

**Published:** 2022-06-13

**Authors:** Eman A. Rabie, Inas S. M. Sayed, Khalda Amr, Hoda A. Ahmed, Mostafa I. Mostafa, Nehal F. Hassib, Heba El-Sayed, Suher K. Zada, Ghada El-Kamah

**Affiliations:** 1 Medical Molecular Genetics Department, Human Genetics & Genome Research Division (HGGR), National Research Centre (NRC), Cairo 12622, Egypt; hoda_radwan80@yahoo.com; 2 Biology Department, School of Sciences and Engineering, The American University in Cairo (AUC), Cairo 11835, Egypt; suzada@aucegypt.edu; 3 Orodental Genetics Department, Human Genetics & Genome Research Division (HGGR), National Research Centre (NRC), Cairo 12622, Egypt; inas_sayed@hotmail.com (I.S.M.S.); mostafanrc@yahoo.com (M.I.M.); nounih@hotmail.com (N.F.H.); 4 Clinical Genetics Department, Human Genetics & Genome Research Division (HGGR), National Research Centre (NRC), Cairo 12622, Egypt; drheba_ahmed@hotmail.com

**Keywords:** *TSPEAR*, ectodermal dysplasia, tooth agenesis, dysmorphic facial features, genetics of North Africa

## Abstract

Ectodermal dysplasia (ED) are hereditary disorders characterized by the disturbance of the ectodermal development of at least two of four ectodermal tissues: teeth, hair, nails and sweat glands. Clinical classification of ED is challenged by overlapping features, variable expressivity, and low number of patients, hindering full phenotypic spectrum identification. Disease-causing variants in elements of major developmental pathways, e.g., Ectodysplasin/NFκB, Wnt, and Tp63 pathways, have been identified in fewer than half of ED phenotypes. Whole-exome sequencing (WES) was performed for ten Egyptian ED patients presenting with tooth agenesis, normal sweating, scalp hypotrichosis, and sharing characteristic facial features. WES was followed by in silico analysis of the effects of novel detected genetic variants on mRNA and protein structure. The study identified four novel rare pathogenic and likely pathogenic *TSPEAR* variants, a gene which was recently found to be involved in ectodermal organogenesis. A novel in-frame deletion recurred in eight patients from six unrelated families. Comparing our cohort to previously reported *TSPEAR* cohorts highlighted the influence of ethnicity on *TSPEAR* phenotypic affection. Our study expands the clinical and mutational spectrum of the growing *TSPEAR* associated phenotypes, and pinpoints the influence of WES and in silico tools on identification of rare disease-causing variants.

## 1. Introduction

Ectodermal dysplasia (ED) is a nosologic group of hereditary disorders of the development and/or homeostasis of two or more tissue derivatives of the human ectoderm [1]. In 1970s, Freire-Maia classified 186 ED phenotypes into two groups: group A, in which at least two of four classical ectodermal derivatives are affected, these being the hair, teeth, nails, and sweat glands, and group B in, which only one of the classical ectodermal derivatives is affected, together with at least one other tissue of ectodermal origin, e.g., central nervous system, melanocytes, adrenal medulla, or lacrimal, Meibomian, mammary and thyroid glands. Eleven subgroups stem from group A according to the affected structures, e.g., hair/teeth/nails/sweat glands, hair/teeth/nails, hair/nails, teeth/sweat glands, etc. [2,3,4,5,6,7]. Concomitant disturbance of tissues originating from other embryonic layers has been reported, e.g., cardiofaciocutaneous syndrome (OMIM #115150) and cleft lip/palate-ED syndrome (OMIM#225060) [1]. A consensus on ED classification remains challenging owing to overlapping features, variable expressivity within the same disorder, allelic disorders, incomplete penetrance, and low number of cases, hindering full phenotypic spectrum identification. Consequently, the Freire-Maia classification system has been dynamically updated to include newly emerging causative genes and syndromes, and exclude single case reports [1,8,9].

Only 75 ED phenotypes have been linked to 77 genes and nine chromosomal regions [8,9]. ED disease-causing variants are usually identified in genes encoding signaling proteins, e.g., Ectodysplasin (Eda), transcription factors, e.g., tumor protein p63 (Tp63), and structural proteins, e.g., cadherins, keratins, and connexins. These proteins are involved in or closely interacting with highly conserved developmental pathways, e.g., ectodysplasin/nuclear factor kappa B (Eda/NFκB), wingless type/β catenin (Wnt/β catenin), bone morphogenic protein (BMP), tumor protein p63 (Tp63), and fibroblast growth factor (FGF) pathways [10]. During embryogenesis, signaling pathways cross-talk at the placodes; the primary sites for ectodermal morphogenesis and organogenesis where epithelial–mesenchymal transition occurs and different ectodermal derivatives arise [11,12]. Several groups have proposed classification of EDs based on their molecular etiologies [13,14,15,16]. Recently, a molecular-based classification system classified different EDs according to the disrupted molecular pathways into five clusters: (1) Eda/NFκB pathway, (2) Wnt pathway, (3) Tp63 pathway, (4) structure group, and (5) other/unknown [1]. Given the current emerging genomic revolution, a consensus on a multi-axis model of classification has been adopted, incorporating phenotypes, modes of inheritance, causative genes and molecular pathways involved [1,17]. Owing to the nature of ED as a nosologic group, the clinical-based classification guides the identification and rapid diagnosis of cases, while gene function-based classification is of importance for genotype-phenotype correlation, future identification of new signaling pathways and the development of therapeutic options [18].

The most common ED phenotype is X-linked hypohidrotic ectodermal dysplasia (XL-HED), also known as Christ–Siemens–Touraine syndrome (OMIM#305100); it accounts for 80% of HED cases and has incidence of 1.6 in 100,000 male births [6,19]. HED is characterized by the classical triad of sparse or absent hair (hypotrichosis/atrichosis), absent or missing teeth (anodontia/hypodontia), and decreased or absent sweating (hypohidrosis/anhidrosis). Distinct facial features of HED patients include: short face, depressed nasal bridge, frontal bossing, everted lips, alveolar bone hypoplasia, saddle nose, and large low set ears [20]. XL-HED is exclusively caused by disease-causing variants of *EDA* gene encoding the ligand ectodysplasin (Eda) of EDA/NFκB pathway, which activates NFκB, the vital transcription factor regulating downstream targets in the ectodermal organogenesis. Autosomal inheritance of HED was attributed to biallelic disease-causing variants of different genes including the Eda receptor (*EDAR*), its associated death domain (*EDARADD*), and wingless-type 10A (*WNT10A*) genes [21]. Of interest is the clinical spectrum of EDA disease-causing variants, which in addition to HED, have been found to also cause non-syndromic isolated tooth agenesis (NSTA) as well as ED without hypohidrosis. This overlapping of phenotypes was also a feature of *WNT10A* disease-causing variants which were identified in NSTA, HED, odontoonychodermal dysplasia (OODD; OMIM#257980) and Schopf–Schulz–Passarge syndrome (OMIM#224750) [21,22,23].

Unlike ED causative genes that have been well phenotypically and molecularly characterized, mutations of *TSPEAR* (OMIM#612920, located in chromosome 21q22.3), the gene encoding Thrombospondin-type laminin G domain and Epilepsy-Associated Repeats (EARs) protein, have been recently reported to cause different autosomal recessive ED phenotypes and NSTA [24]. The 669-amino-acid-long Tspear protein is predicted to harbor two protein-interacting functional domains: the N-terminal thrombospondin-like laminin G domain and the C-terminal-seven EARs domain [25,26]. A homozygous frameshift *TSPEAR* variant (c.1726_1728delGTCinsTT; p.(Val576Leufs*38)) was first reported to cause sensorineural hearing loss (SNHL) in three siblings from one Iranian consanguineous family who had no ED features [25]. However, subsequent reports identified the same *TSPEAR* (c.1726_1728delGTCinsTT; p.(Val576Leufs*38)) variant in three patients: two ED patients and one NSTA patient all presented with normal hearing [27,28,29]. Among a total of 22 previously reported unrelated families with probands harboring biallelic *TSPEAR* disease-causing variants, the majority of *TSPEAR* associated phenotypes fall within ED or TA spectra [24,29].

We identified four rare pathogenic and likely pathogenic *TSPEAR* variants in a North African cohort of ten Egyptian ED patients descending from eight unrelated families. Our patients were initially found to be negative for *EDA*, *EDAR*, *EDARADD*, and *WNT10A* disease-causing variants, and thus were opted for whole exome sequencing (WES). Patients presented with TA, scalp hypotrichosis, normal sweating, and shared common characteristic facial features. Our study expands the clinical and mutational spectrum of *TSPEAR* associated phenotypes, and highlights the power of WES in identification of rare disease-causing variants.

## 2. Materials and Methods

### 2.1. Ethical Aspects

All patients, parents, legal guardians, and available family members signed written informed consents to be included in our study after thorough explanation and discussion. Study design and ethical procedures were approved by the institutional review boards of the National Research Centre (NRC) of Egypt and the of the American University in Cairo (AUC) according to the Declaration of Helsinki. Patients were recruited from the Genodermatoses and Oro-Dental Genetics clinics of the Medical Research Excellence Centre, NRC, Egypt.

### 2.2. Patient Phenotyping and Inclusion Criteria

Patients were included based on: (1) clinical diagnosis of ED in which at least two ectodermal tissue derivatives (hair, nails, teeth and skin) were impaired, or diagnosis of NSTA; and (2) previous molecular exclusion of *EDA*, *EDAR*, *EDARADD*, and *WNT10A* disease-causing variants using targeted next generation sequencing (NGS) panel. Detailed medical and family histories were recorded, and three generation pedigrees were constructed. Thorough medical examination of the patients was performed with special emphasis on skin, hair, nails and other ectodermal elements. Hypohidrosis was assessed by patients’ reports of unexplained fever attacks that are unresponsive to antipyretics, flushing with warm temperature, and intolerance to hot temperatures (35–40 °C), as well as clinical examination of perspiration patterns with emphasis on armpits, palms, soles, and other focal areas (craniofacial, buttocks, back, groin, and breasts).

Detailed oral and dental examination of the patients and parents was performed. Panoramic dental radiographs were used to assess dental phenotype. Oligodontia describes the agenesis of six teeth or more while hypodontia describes the agenesis of fewer than six teeth, excluding third molars [30]. Clinical evaluation ensured that patients met the inclusion criteria for ED and differentiated between syndromic and NSTA. Other associated dental anomalies, such as conical teeth, were also recorded. Conical teeth denote teeth exhibiting a sharply pointed crown or incisal edge [31]. All patients were referred for hearing loss testing.

### 2.3. Molecular Analyses

#### 2.3.1. DNA Extraction and Whole Exome Sequencing (WES)

Genomic DNA was extracted from peripheral blood samples of all participants using QIAamp DNA Mini Kit (Qiagen, Hilden, Germany). Quality and quantity of DNA samples of patients were assessed using fluorometric Denovix Qubit™ dsDNA BR Assay Kit (ThermoFisher, Waltham, MA, USA). DNA samples from 10 patients were sequenced using the Twist Human Core Exome Plus kit (Twist Bioscience, San Francisco, CA, USA) and NovaSeq 6000 system (Illumina, San Diego, CA, USA) according to the manufacturer’s protocol. Libraries were prepared in paired-end mode (2 × 100 bp) for an output of 6 GB per sample, and an average coverage of 50X. Sequencing reads were demultiplexed using Illumina bcl2fastq (2.20) and adapter sequences were trimmed using Skewer (version 0.2.2) [32]. The quality of the generated FASTQ files was analyzed with FastQC software (version 0.11.5; Illumina, San Diego, CA, USA). BAM files were generated using Burrows Wheeler Aligner (BWA) by aligning reads against the GRCh37/hg19 reference genome. Variant calling files were generated using previously described pipeline [33].

#### 2.3.2. Variant Annotation and Filtration

PhenoDB tool was used to annotate Vcf files using ANNOVAR [34,35]. Variants were filtered based on depth of coverage and minor allele frequencies (MAF) (less than 1% MAF) in large population databases, including dbSNP [36], 1000 Genomes Project [37], and the Genome Aggregation Database (gnomAD v2.1.1) [38]. Prioritization of the filtered variants was performed based on the following considerations: (1) mode of inheritance, e.g., homozygous and compound heterozygous, i.e., biallelic variants in case of recessive traits, (2) gene function, ontology, pathways involved and associated disease(s), and (3) variant type and predicted deleterious effects using different in silico prediction tools. Tools targeting evolutionary conservation scores include SIFT (Sort Intolerant From Tolerant) [39], Mutation Assessor [40], CADD (Combined Annotation Dependent Depletion) [41] and GERP (Genomic Evolutionary Rate Profiling) [42], while those targeting evolutionary conservation plus protein structural domains include Polyphen-2 [43], MutationTaster2 [44], and PROVEAN (Protein Variation Effect Analyzer) [45]. The NMDEscPredictor tool was used to predict the likelihood of nonsense-mediated decay (NMD) in the case of frameshift variants [46].

#### 2.3.3. Variant Segregation

Sanger sequencing was used to confirm that prioritized variants segregated consistently among parents and available family members according to the predicted mode of inheritance. We designed primers targeting *TSPEAR* exons which harbor the filtered variants of interest using Primer3 tool [47], Table 1. PCR was carried out as previously described [48]. Reactions were sequenced according to manufacturer’s recommendation using the Big Dye Termination kit (Applied Biosystems, Waltham, MA, USA), and ABI Prism 3500 Genetic Analyzer (Applied Biosystems, Waltham, MA, USA). Variants were named based on Human Genome Variation Society nomenclature recommendations [49]. The standards of the American College of Medical Genetics and Genomics (ACMG) were used to classify the level of variant pathogenicity, i.e., pathogenic, likely pathogenic, variant of unknown significance (VUS), benign, or likely benign [50].

#### 2.3.4. In Silico Prediction of Protein Structural Alterations Caused by *TSPEAR* Variants

In silico tools were used to predict the potential impact of the missense variants on Tspear structure. Since Tspear had no PDB (Protein Data Bank) crystal structure, we retrieved a predicted protein structure model covering the entire Tspear amino acid chain generated by AlphaFold [51] from the Uniprot database (Uniprot ID: Q8WU66) [52]. Two different in silico tools were used: PREMPS and Missense3D [53,54]. PremPS predicts the effect of missense variants on protein stability via calculation of the change in the unfolding free energy (ΔΔG) for each mutated protein, i.e., the changes in Gibbs free energy between folded and unfolded states of the protein. PREMPS also shows the location of the mutated residue, i.e., on the surface or core of the protein, and provide the predicted 3D structure of the mutated protein with emphasis on the changes in the types of bonding at the mutated residue [53]. Missense3D predicts the mutated protein 3D structure to identify changes in conformational features including changes in cavity volume, buried versus exposed states of target residue, changes in charge and hydrophobicity, and changes in relative solvent accessibility (RSA) [54].

### 2.4. Comparison of Phenotypic Variabilities among TSPEAR Cohorts in Relation to Ethnicities

We compared clinical phenotypes and dysmorphic facial features of our studied cohort of patients to previously published cases that were similarly identified to harbor biallelic *TSPEAR* disease causing variants [24,25,27,28,29,55,56]. Patients were grouped according to their reported ethnicities into: North African, Middle Eastern, European and others. The involvement of different ectodermal elements in ED phenotype of these cases was also compared. Data are summarized as frequencies and percentages for categorical variables. Chi-squared test for independence (χ^2^) was used to compare each clinical feature across different ethnicities. Statistical analyses were performed using IBM SPSS Statistics for Windows, version 19.0. (IBM Corporation, Armonk, NY, USA). *p*-values < 0.001 were considered statistically significant.

## 3. Results

### 3.1. Clinical Features

**Figure 1:**
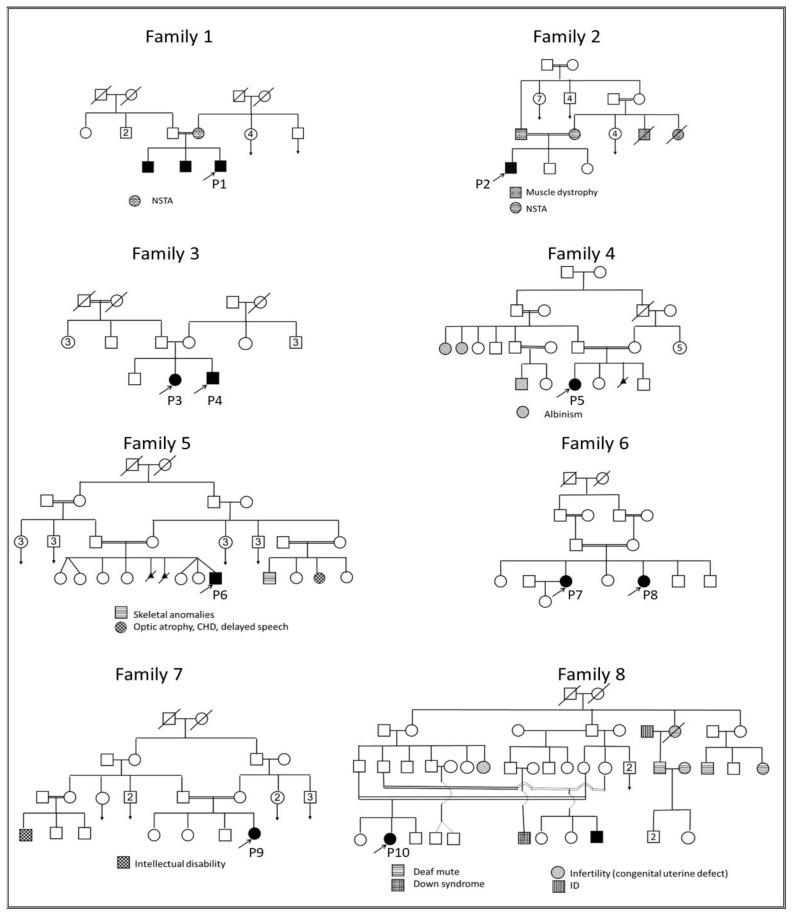
Pedigrees of the ten ED patients (P1–P10). Parental consanguinity is evident in all families except family 3. The presence of other genetic disorders is highlighted under each pedigree. The mother of P1 and both parents of P2 had hypodontia (i.e., non-syndromic isolated tooth agenesis; NSTA). Squares refer to males, circles refer to females, and triangles refer to miscarriages. Open shapes refer to unaffected family members, closed shapes refer to affected family members, and deceased family members are denoted by diagonal lines across their shapes. Probands are denoted by arrows and designated the numbering (P1–P10). Double relationship lines refer to consanguinity. Abbreviations: CHD: congenital heart disease, ID: intellectual disability, and NSTA: non-syndromic tooth agenesis.

**Figure 2:**
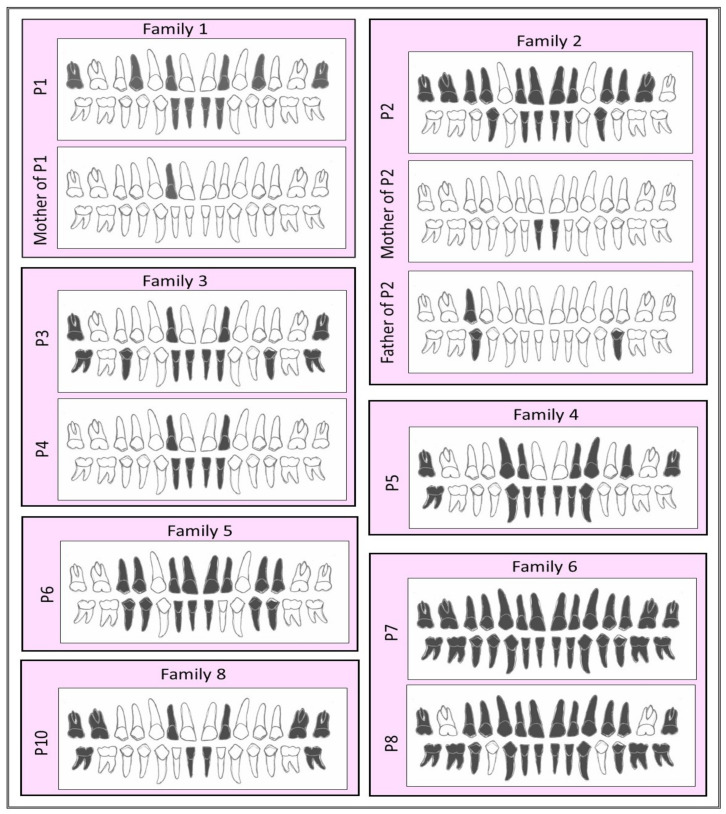
Schematic representation of the missing teeth for different patients. White tooth color denotes present teeth, and black denotes congenitally missing teeth. All patients showed oligodontia except P4, who had hypodontia. The number of missing teeth in P2 is shown in comparison to his heterozygous parents, who showed hypodontia. The number of missing teeth could not be assessed in P9 due to her young age and the impossibility of performing a panoramic radiograph. For panoramic radiographs, see Supplementary Figures.

**Figure 3:**
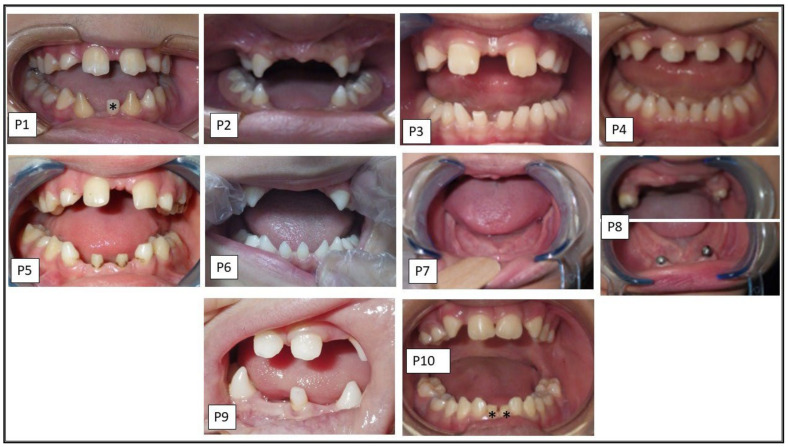
Intraoral photographs of ED patients (P1–P10). The figure shows conical teeth in P1–P6, P9 and P10 and retained deciduous teeth (indicated by asterisks) in P1 and P10.

Ten patients descending from eight unrelated families of Egyptian origin were recruited. All families showed parental consanguinity except family 3, see Figure 1. Clinical data are summarized in Table 2. Patients showed normal sweating except P5, who had hyperhidrosis of palms and soles. The involvement of teeth was consistent among the entire cohort in the form of oligodontia except P7, who showed total agenesis of all teeth, see Figure 2. Conical-shaped anterior teeth were observed in all patients with the exception of P7, as she was completely edentulous, and P8, as she lacks any anterior teeth. Retained deciduous teeth and delayed eruption of teeth were found in two patients (P1 and P10), see Figure 3. None of the parents and family members showed ED-related features except the mother of P1 and the parents of P2, who had hypodontia milder than their respective probands or any other probands, see Figure 2.

**Figure 4:**
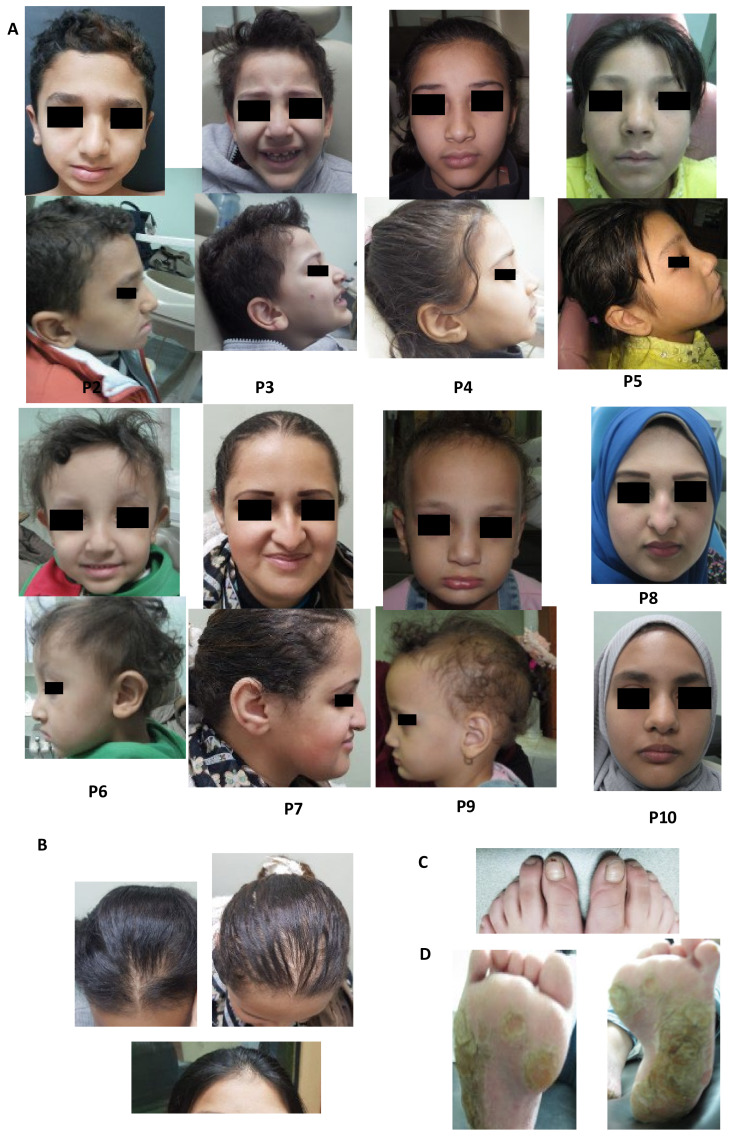
Phenotypic features of the *TSPEAR*-ED cohort. (**A**) Facial photographs of P2–P10 showing common characteristic facial features including broad forehead, short philtrum, prominent and broad nasal root, broad nose, low set ears, and thick lips. Malar hypoplasia can be observed in P2, P4, P6 and P7. (**B**) Scalp hypotrichosis was more prominent on the anterior part of the scalp. (**C**) Dysplastic nails. (**D**) Severe keratoderma observed in P1. P1 refused to be photographed, and P8 and P10 opted to keep their hair covered.

Patients showed common characteristic facial features: broad forehead, short philtrum, prominent and broad nasal root, broad nose, low set ears, and thick lips, see Figure 4A. Thick and everted lips, particularly the lower lip, were common oral findings: thick lower lips were found in eight patients and everted lower lips were also observed in eight patients, see Table 2. Some patients had malar hypoplasia (P2, P4, P6 and P7), see Figure 4A. Common hair-related features were scalp hypotrichosis, more prominent on the anterior part of the scalp, and high anterior hairline, except for P2, who had a normal hairline, and density, see Figure 4A,B. Five patients had hypotrichosis or atrichia of eyebrows (P5–P9). Dysplastic nails were observed in four patients (P1, P2, P8 and P9), see Figure 4C. Hyperkeratotic skin was observed in two patients (P1 and P5), and P1 had severe palmoplantar keratoderma, see Figure 4D. Uncommon findings were skeletal abnormalities in P1, delayed motor and mental milestones in P6, and decreased salivary flow in P7.

### 3.2. Molecular Data

#### 3.2.1. *TSPEAR* Variants’ Identification, Segregation and MAF

**Figure 5:**
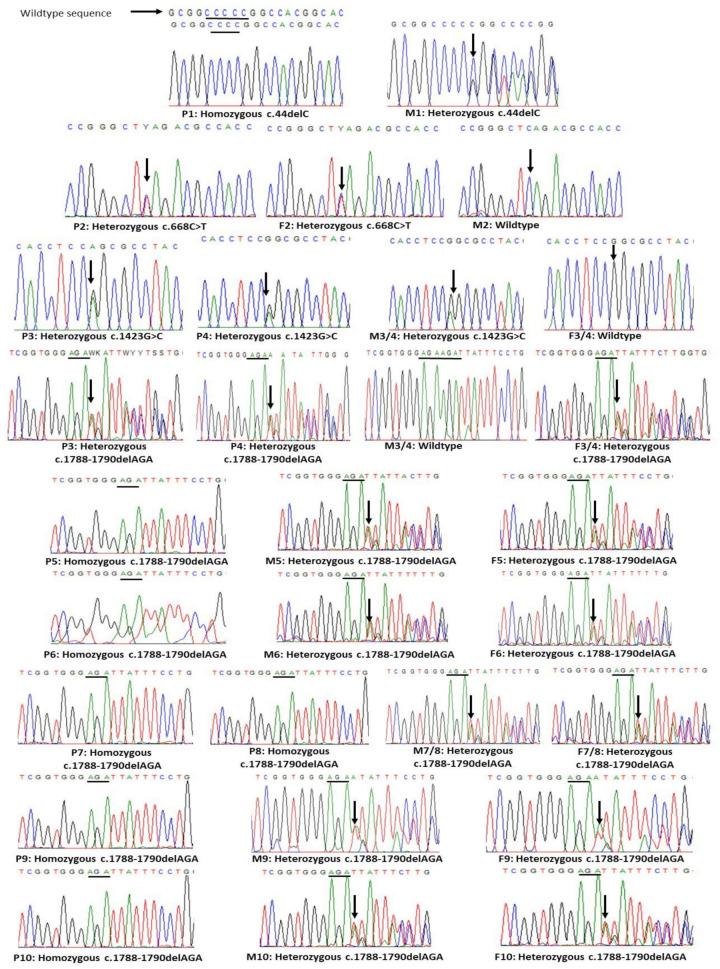
Segregation analysis of *TSPEAR* variants. The figure features the Sanger sequencing chromatograms of ten patients (P1–P10), their fathers (F2–F10), and their mothers (M1–M10). Please note that parents are assigned the same numbers as their corresponding proband/s. Variants of *TSPEAR* (NM_144991.3) are designated under each chromatogram.

**Figure 6:**
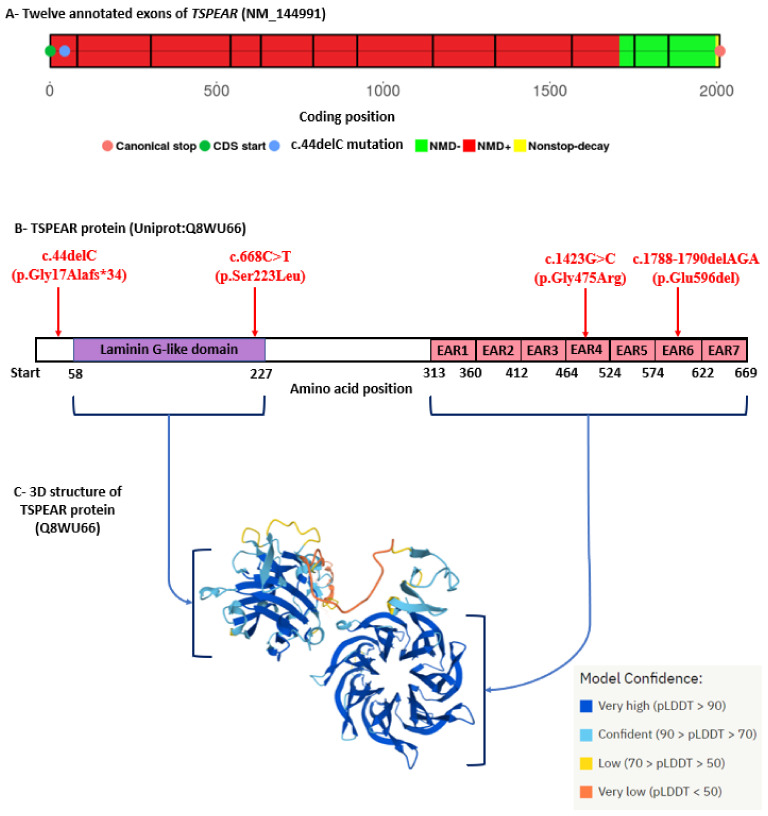
Schematic representation of *TSPEAR* transcript, protein domains and 3D structure. (**A**) prediction of nonsense-mediated decay (NMD) for the *TSPEAR* (NM_144991) c.44delC variant using the NMDEscPredictor tool. The c.44delC variant is shown to be located in NMD susceptible region (NMD+, red colored) of the *TSPEAR* transcript. (**B**) Schematic diagram of Tspear protein (Uniprot:Q8WU66); amino acid positions were retrieved from the Uniprot database. The locations of the four identified *TSPEAR* variants relative to the thrombospondin-type laminin G domain (purple box) and the seven Epilepsy-Associated Repeats (EARs) (pink boxes) are shown. (**C**) The predicted 3D structure of the Tspear protein (created by AlphaFold and retrieved from Uniprot database). Blue arrows in (**B**) refer to laminin G domain and EARs domains in the 3D structure. The color-coded per-residue model confidence score (pLDDT) is shown to be between 0 and 100.

Exome analyses of the ten studied ED patients identified four different novel *TSPEAR* variants (NM_144991.3, NP_659428.2), see Figure 5. P1 had a homozygous frameshift deletion (c.44delC; p.(Gly17Alafs*34)) in exon 1 that substitutes glycine residue with alanine and is predicted to create a frameshift of 33 amino acids. The resulting mRNA is predicted to undergo NMD by NMDEscPredictor tool [46], see Figure 6A. Variant segregation identified the heterozygous form of c.44delC in mother of P1, see Figure 5. The variant was not found in any of the large population databases (dbSNP, 1000G and gnomAD), nor in our in-house database of 55 Egyptian exomes. According to ACMG guidelines, c.44delC is classified as a “pathogenic” variant, see Table 3.

P2 had a heterozygous missense variant (c.668C>T; p.(Ser223Leu)) in exon 5 substituting serine for leucine. The variant was inherited from the father, who carries the same heterozygous missense variant, unlike the wildtype mother, see Figure 5. Exome analysis of P2 neither identified other variants in *TSPEAR* nor in other ED-related genes. MAF of c.668C>T are less than 0.1% in dbSNP, 1000G, and gnomAD, and the minor allele was not found in our in-house database. The variant is classified as “likely pathogenic” according to ACMG guidelines, see Table 3.

P3 and P4 are affected siblings, both of whom had compound heterozygous *TSPEAR* variants: a missense (c.1423G>C; p.(Gly475Arg)) in exon 9 and an in-frame deletion (c.1788-1790delAGA; p.(Glu596del)) in exon 11. Segregation analysis showed that the missense allele was maternally inherited while the in-frame deletion allele was paternally inherited, see Figure 5. The same in-frame deletion c.1788-1790delAGA (p.Glu596del) was identified in six patients (P5 to P10) in homozygous form and variant segregation confirmed germline inheritance in their families, see Figure 5. MAF for c.1788-1790delAGA are less than 0.026% in dbSNP, and gnomAD, and the minor allele was found in neither the 1000G database nor in our in-house database. The missense c.1423G>C variant has MAF of less than 0.0007% in gnomAD, and the minor allele was not reported in dbSNP, 1000G, or in our in-house database. Both c.1788-1790delAGA and c.1423G>C are classified according to ACMG guidelines as “pathogenic” and “likely pathogenic” variants, respectively, see Table 3.

The four identified *TSPEAR* variants are located in highly conserved regions in functional protein domains, see Figure 6B. This was also evident in their positive GERP scores, which refers to constrained genomic regions across different species, see Table 3. Moreover, the average CADD score for the two missense variants (c.668C>T and c.1423G>C) was approximately equal to 25, which predicts these variants to be among the top 1% of the most deleterious substitutions of the human genome, see Table 3. Both c.668C>T and c.1423G>C variants are reported in ClinVar; however, no clinical criteria/phenotypes are provided for their entries.

#### 3.2.2. In Silico Predicted Protein Structural Alterations of Missense *TSPEAR* Variants

**Figure 7:**
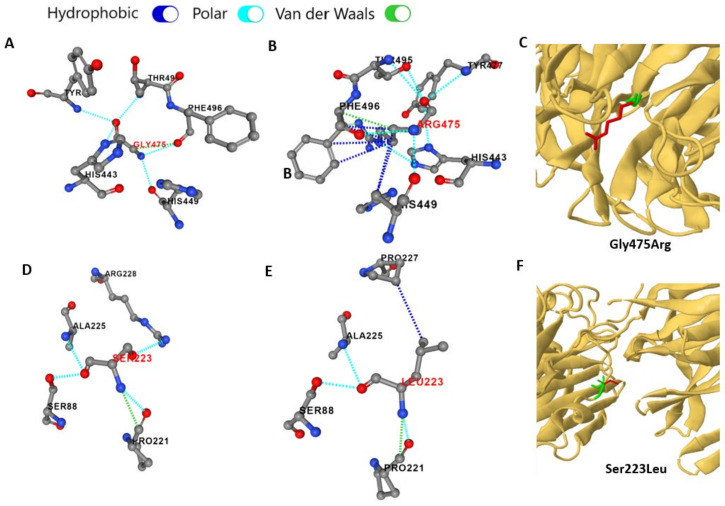
In silico predicted protein structural alterations of the two missense *TSPEAR* variants (c.1423G>C; p.(Gly475Arg)) and (c.668C>T; p.(Ser223Leu)). The color code of the non-covalent interactions is shown at the top of the figure. The PremPS tool predicted that the 3D structure changes from wildtype Gly475 residue (**A**) to the mutant Arg475 (**B**). (**C**) The Missense 3D tool shows the difference between the side chain of wildtype Gly475 (green) and mutated Arg475 (red). Similarly, the PremPS tool predicted that the 3D structure changes from the wildtype Ser223 residue (**D**) to mutant Leu223 (**E**). (**F**) The Missense 3D tool shows the difference between the side chain of wildtype Ser223 (green) and the mutated Leu223 (red).

The PremPS tool predicted that the two missense (c.1423G>C; p.(Gly475Arg)) and (c.668C>T; p.(Ser223Leu)) variants are destabilizing, with ∆∆G values of 0.53 and 0.9 kcal/mol, respectively. For the (c.1423G>C; p.(Gly475Arg)) variant, the wildtype uncharged Gly475 residue has no side chain, is buried in the protein core, and interacts with His443, His449, Tyr477, Thr495, and Phe496 via polar interactions, see Figure 7A. The mutant Arg475 residue introduces destabilizing conformational changes via its long positively charged side chain; the long aliphatic side chain forms multiple hydrophobic interactions, particularly with His449 and Phe496, and its positively charged amino groups result in increased polar interactions, see Figure 7B,C. Missense 3D software predicted Gly475Arg mutation to increase relative solvent accessibility (RSA) from 0 to 22%, i.e., the buried glycine switches to an exposed arginine, with consequential damaging conformational changes. For the (c.668C>T; p.(Ser223Leu)) variant, the change from wildtype polar Ser223 residue, which is located on the protein surface, to the mutant non-polar Leu223 is predicted to cause loss of polar interaction with Arg228 and the gain of a new hydrophobic interaction with Pro227, thus destabilizing the protein, see Figure 7D–F. Missense 3D predicted a change in RSA from 27.6% in the case of Ser223 to 53.6% for Leu223, and the expansion of the surface pocket by 41.7 Å^3^; however, predictions did not pass the software’s criteria for structural damage (expansion/contraction of the surface pocket volume of ≥ 70 Å^3^, and change in exposed/buried amino acid state where RSA is less than 9% for buried residue and difference in RSA is at least 5%).

### 3.3. Comparison of Clinical Phenotypes and Dysmorphic Facial Features of TSPEAR Cohorts of Different Ethnicities

The clinical features of our *TSPEAR*-ED cohort (ten patients from eight unrelated families) of Egyptian, i.e., North African origin are detailed in comparison to 28 previously published *TSPEAR* cases from 22 unrelated families of different ethnicities, see Table 4. Phenotypes associated with biallelic *TSPEAR* variants can be categorized into: (1) ED with or without TA (57.9%); (2) TA without other ectodermal features (e.g., isolated TA or TA with other non-ED-related features) (21.1%); (3) SNHL without ED features (18.4%); and (4) SNHL with ED, which was reported in only one patient (2.6%). Only four previously reported patients had ED without TA; however, they had conical-shaped teeth, which does not fulfill the criteria of TA; still, it suggests the involvement of teeth [24]. Clinical presentation of dysmorphic facial features showed statistically high significant dependence (*p* = 0.0001) on ethnic origin. North African as well as Middle Eastern patients were reported to have characteristic dysmorphic facial features in contrast to patients of European origin. The details of these dysmorphic features are summarized in Table 4. The involvement of different ectodermal elements in a total of 24 *TSPEAR*-ED cases showed that teeth were the most affected ectodermal derivative (82.6%), followed by the hair (78.3%), nails (43.5%) and sweat glands (39.1%), see Table 4.

## 4. Discussion

Our study expanded the limited reports of *TSPEAR* variants by adding ten patients descending from eight unrelated Egyptian families to the previously published 28 probands from 22 unrelated families of different ethnicities [24,25,27,28,29,55,56]. All of our ten reported *TSPEAR*-ED patients harbored novel *TSPEAR* variants, and they all presented with TA, scalp hypotrichosis, normal sweating, and common characteristic facial features. Similar to the majority of reported cases with biallelic *TSPEAR* variants, all of our patients had normal hearing, which did not support the disputed association between *TSPEAR* and SNHL [57]. Four of seven previously reported *TSPEAR*-SNHL cases were identified to have mutations in other hearing-loss-associated genes, e.g., *GJB2*, *GJB6*, *TMPRSS3*, and *SLC26A4* [24,58]. The other three *TSPEAR*-SNHL cases were three siblings from one family in which a homozygous frameshift *TSPEAR* variant segregated with SNHL [25]. The hearing loss expert panel of ClinGen database cited this family as the source of conflicting evidence regarding *TSPEAR* and SNHL association, since the same *TSPEAR* variant was identified in two ED families and one NSTA family with normal hearing [24,25,59]. It was suggested that the SNHL phenotype might have reduced penetrance or that *TSPEAR* variants might not be the monogenic cause of SNHL owing to the locus heterogeneity of SNHL [24].

Our ten reported *TSPEAR*-ED patients shared characteristic dysmorphic facial features: broad forehead, short philtrum, prominent and broad nasal root, broad nose, low set ears, and thick and everted lips. We showed that *TSPEAR*-associated dysmorphic facial features vary according to ethnic origin (*p* = 0.0001), with an emphasis on patients from North African or Middle Eastern origin. The influence of ethnicity on disease phenotype has been described before in rare and common disorders [60,61]. Our reported facies were distinguishable from previously reported patients from other ethnicities, see Table 4. Some features were shared with patients from Middle Eastern origin, e.g., scalp hypotrichosis, which was more prominent on the anterior of the scalp in six of our ten reported *TSPEAR*-ED patients, has similarly been reported in three Palestinian patients [27]. Everted lips were common oral finding in our patients. Bondarets and McDonald [62] reported that everted lips are characteristic feature of HED and that tooth agenesis results in poor development of the alveolar ridge with a consequent reduction in lower facial height in both HED and NSTA. The marked reduction in facial height was severe enough in HED to cause everted lips, but not in isolated tooth agenesis. Retained primary teeth and delayed dental development seen in patients P1 and P10 were among dental anomalies that have previously been reported to be found in association with tooth agenesis [63,64].

The involvement of different ectodermal elements in the *TSPEAR*-ED phenotype showed that teeth and hair are affected at higher frequency than nails and sweat glands, see Table 4. The involvement of *TSPEAR* in both hair and teeth ontogenesis was found to be mediated functionally via Notch signaling, particularly Notch1 [27]. Notch1 is one of the four transmembrane receptors of the highly conserved Notch signaling pathway that is essential for deciding cellular fate via cell-to-cell communication during embryogenesis, and also in maintenance of homeostasis in adulthood [65,66]. The extracellular compartment of Notch receptors transduces cell-to-cell signals by interacting with transmembrane ligands of neighboring cells, e.g., Delta-like and Jagged ligands. Ligand–receptor binding results in proteolytic cleavage of the Notch receptor’s intracellular domain (NICD), which translocates to the nucleus, forming a ternary protein complex with the DNA-binding factor (RBP-J) and Mastermindlike-1 (MAML) necessary for downstream transcriptional regulation of target genes [67]. Microarray expression data of primary human keratinocytes following *TSPEAR* knock down showed down-expression of *NOTCH1*, and altered expression of genes encoding proteins within *NOTCH1* interactome, e.g., *CDCA7*, *DLL1*, *IGFBP3*, and *TP63*, and/or genes encoding proteins involved in hair and tooth development, e.g., *KRT1*, *KRT10* and *WNT10A* [27]. Further evidence of *TSPEAR*–*NOTCH1*-mediated function was down-expression of *NOTCH1* in the epidermis of a skin biopsy obtained from an ED patient bearing the homozygous *TSPEAR* loss of function (c.1726_ 1728delGTCinsTT, p.(Val576Leufs*38)) variant [27]. It is unknown how Tspear and Notch1 proteins interact or if their interaction is mediated by proteins involved in previously identified ED pathways; thus, the *TSPEAR*-ED phenotype might prospectively fit into one of the classified ED pathway clusters or propose an additional cluster.

In mice, Tspear is expressed in the enamel, as well as different compartments of the murine hair follicle, e.g., hair bulb, shaft, outer and inner sheaths. Knock down of murine *TSPEAR* in vitro hair follicle culture decreased hair bulb size, arrested hair growth, increased apoptosis, and reduced Notch1 expression [27]. Murine models support the critical role of Notch signaling in dental stem cell differentiation and enamel formation throughout early embryogenesis [27,68]. On the other hand, Notch signaling was found to be dispensable for initial placodal formation and hair follicle differentiation but indispensable for subsequent cellular differentiation postnatally [69,70,71,72]. The critical role of Notch signaling in tooth and hair development might explain the higher occurrence of teeth and hair manifestations in the *TSPEAR*-ED phenotype.

While *TSPEAR* expression in nails, sweat and salivary glands has not been studied, Notch signaling is involved in their organogeneses in mice. Downregulation of Notch1 directs sweat gland differentiation, while its upregulation is required for salivary glands’ differentiation and growth and promoting mitosis in the nail matrix [73,74,75]. The ten reported patients herein had ED without hypohidrosis, but only one patient showed decreased salivary flow, which has not been reported in *TSPEAR*-ED cases before. Relatively lower occurrence of sweat gland involvement in *TSPEAR*-ED cases might be attributed to the differences in the roles of Notch signaling in these tissues or the differences in the expression or functional role of *TSPEAR* in their ontogeneses.

Tspear is predicted to function via its two evolutionarily conserved domains, the thrombospondin-like laminin G domain and the seven EARs domain. Based on sequence similarity, Tspear is considered to be apart of a protein superfamily comprising EARs, which form a seven-bladed β propeller domain that presumably acts as a ligand binding domain, see Figure 6C [25,26,76]. Approximately 53% of the 30 *TSPEAR* disease-causing variants, including our four identified *TSPEAR* variants, are missense variants, which are predicted to affect one of these Tspear domains [77]. Other types of *TSPEAR* disease-causing variants include small frameshift deletions predicted to produce mRNA that is NMD susceptible, including the novel (c.44delC; p.(Gly17Alafs*34)) variant identified in ED patient (P1). Two previously reported small frameshift deletions featuring the ED phenotype, (c.38delT; p.(Leu13Argfs*38)) and (c.454_457delCTGG; p.(Leu152Trpfs*29)), were NMD susceptible, while the (c.1505delA; p.(Lys502Argfs*67)) variant, featuring TA, was predicted to escape NMD [24,27].

Interestingly, the mother of P1, a heterozygous carrier of the (c.44delC; p.(Gly17Alafs*34)) variant, showed mild hypodontia in the form of a missing upper later incisor, see Figure 2. Mild phenotypic features of heterozygous carriers of recessive alleles have previously been reported in carrier parents of ED patients harboring *EDAR* disease-causing variants [48,78], and similarly in a carrier father of a TA patient who carried a *TSPEAR* missense (c.1877T>C; p.(Phe626Ser)) variant [28]. Still, there is still the possibility that these parents carry a deep intronic *TSPEAR* variant, or alternatively, a variant of another TA-causing gene. In another proband, P2, a heterozygous likely pathogenic *TSPEAR* missense variant (c.668C>T; p.(Ser223Leu)) was identified as being paternally inherited. The heterozygous carrier father had hypodontia milder than that of P2, but with no other ED-related features, see Figure 2. The second allele responsible for the ED phenotype could not be identified in P2, and we suspect that the second allele might be outside the WES capture region, e.g., non-coding or deep intronic variant, which necessitates the whole-genome or RNA sequencing methods [79,80,81]. The second allele may be maternally inherited, since the mother of P2 also had mild hypodontia, see Figure 2.

We identified a novel in-frame deletion (c.1788-1790delAGA; p.(Glu596del)), affecting the sixth EAR domain of Tspear through deletion of its 596th glutamic acid residue. This is the first in-frame deletion to be reported for *TSPEAR*; it was identified in homozygous form in six patients, and in heterozygous form in two other patients. The heterozygous c.1788-1790delAGA allele was found in trans with another *TSPEAR* novel missense variant (c.1423G>C; p.(Gly475Arg)) affecting the fourth EAR of Tspear, see Figure 6B. Having a relatively large number of phenotypically characterized ED patients from the same ethnic origin harboring the same disease-causing variant might propose a founder effect for c.1788-1790delAGA variant. We have previously shown that the HED mutation spectrum in Egyptians was different from that in other studied cohorts, whether this was due to the genes responsible for the phenotype or the incidence of founder mutations owing to high percentage of consanguineous marriages (~33–35%) among Egyptians [48,82,83]. Moreover, the recurrence of the c.1788-1790delAGA variant in eight ED patients shows the phenotypic heterogeneity of that variant. For example, variable patterns of TA were evident, as P7 had total anodontia (28 missing teeth) in contrast to P10, who had ten missing teeth.

Our four reported *TSPEAR* variants were found to be rare and located at conserved genomic locations by mining large population and in-house databases, and reviewing GERP and CADD scores, respectively [41,42]. For missense variants, recent advances in protein modelling and machine learning have allowed in silico variant visualization and analysis of the two missense *TSPEAR* variants, (c.1423G>C; p.(Gly475Arg)) and (c.668C>T; p.(Ser223Leu)). The predicted changes in the protein folding energy (∆∆G values>0.5), as well as the predicted changes in the types of non-covalent bonding between the amino acid residue of interest and the surrounding residues, were evident of destabilization of the mutant proteins. The damaging effect of (c.1423G>C; p.(Gly475Arg)) variant on the Tspear protein conformation is predicted to be more severe than (c.668C>T; p.(Ser223Leu)) variant which can be deduced given the greater difference in size, charge and polarity between glycine and arginine compared to the difference in size and polarity between serine and leucine.

## 5. Conclusions

We expanded the *TSPEAR* mutational spectrum by identifying four novel variants in *TSPEAR* in ten Egyptian patients featuring TA, scalp hypotrichosis, normal sweating, and characteristic facial features, confirming and expanding the clinical distinction of *TSPEAR*-ED as an entity, as well as concluding the role ethnicity plays in *TSPEAR* phenotypic spectrum. Our results do not support the association between *TSPEAR* and SNHL, and show evidence of complete penetrance of TA in our cohort in contrast to other ectodermal elements. We underscore the power of WES for broadening the molecular spectrum of unidentified ED cases. Nonetheless, WES is limited by its capture region, and in some cases whole-genome sequencing or RNA sequencing might be useful in the identification of deep intronic and non-coding variants. Furthermore, functional characterization of Tspear protein-protein interactions could help in the delineation of the mutational and clinical *TSPEAR* profiles.

## Figures and Tables

**Table 1:** Primer sequences used for variant segregation targeting *TSPEAR* exons.

Exon Number	Forward (F) and Reverse (R) Primers Sequences (5′ to 3′)	Annealing Temperature (°C)
Exon 1	F-ACCTCTGTCCCCGCCTTAG	61
	R-CCATCTCCACAGGGTGCTAC	
Exon 5	F-AAGCTCAGTGGTCGCCTCC	62
	R-ACACGAGAGGGGCTGAGAG	
Exon 9	F-TGGGAATAGCACCTGTGATG	59.5
	R-AGAGCAGCACTAGGTTTGGC	
Exon11	F-CCCCGGCTCCTCCTCTATAA	61.5
	R-CCTCGGCAGCTCATTACCT	

**Table 2:** Clinical features of *TSPEAR*-ED cohort.

Fa	P	Consanguinity	Sex	Age	Sweating	Hair	Skin	Nails	Teeth	Conical Shaped Teeth	Other Oral Features	Others	Genotype /Variant	Affected Ectodermal Organs
1	1	+	M	16 y	N	High anterior hairline	Dry skin, severe palmoplanter hyperkeratosis, and keratosis pilaris.	D	O	+	Thick lips, everted lips, macroglossia, median grooved tongue, broad uvula, enamel hypocalcification, retained deciduous teeth, and delayed eruption.	Skeletal abnormalities: talipes equinovarus, pes cavus, low inserted third toes, clinodactyly (toes), arachnodactyly (hands), and no ejaculation. Mother has hypodontia.	Homozygous/ c.44delC p.(Gly17Alafs*34)	Hair, teeth and nails
2	2	+	M	10 y	N	N	Dry palms	D	O	+	Thick lower lip, everted lower lip, macrostomia, short broad philtrum, and very narrow V shaped palate.	Both parents have hypodontia	Heterozygous/ c.668C>T p.(Ser223Leu)	Teeth and nails
3	3	-	F	9 y 6 m	N	Sparse scalp hair *, and high anterior hairline	N	N	O	+	Thick lips, everted lips, deep labiomental sulcus, and dimpled chin.		Compound heterozygous/ c.[1423G>C]; [1788-1790delAGA] p.([Gly475Arg];[Glu596del])	Hair and teeth
3	4	-	M	6 y	N	Sparse scalp hair *, and high anterior hairline	N	N	O	+	Thick lower lip, and everted lips.			Hair and teeth
4	5	+	F	8 y	Hyperhidrosis of palms and soles	Sparse scalp hair *, high anterior hairline and sparse eyebrows	Hyperkeratosis	N	O	+	Thick lips, prominent philtrum, lower pseudolabial cleft, highly attached upper labial frenum, long uvula, and wide overjet.		Homozygous/ c.1788-1790delAGAp.(Glu596del)	Hair, teeth and sweat glands
5	6	+	M	3 y 8 m	N	Sparse scalp hair, high anterior hairline, and sparse eyebrows.	N	N	O	+	Everted lower lip, and bow shape upper lip.	Delayed motor and mental milestones	Homozygous/ c.1788-1790delAGA p.(Glu596del)	Hair and teeth
6	7	+	F	25 y	N	Sparse scalp hair *, high anterior hairline, and sparse eyebrows.	N	N	O	N/A (completely edentulous)	Thick lips, everted lower lip, short philtrum, mandibular prognathism, fissured tongue, and decreased salivary flow rate.		Homozygous/ c.1788-1790delAGA p.(Glu596del)	Hair and teeth
6	8	+	F	21 y	N	Sparse scalp hair *, high anterior hairline, and absent eyebrows.	N	D	O	N/A (missing all anterior teeth)	Short philtrum, and thick lower lip.	Familial Mediterranean fever, and bilateral syndactyly between 2nd and 3rd toes.	Homozygous/ c.1788-1790delAGA p.(Glu596del)	Hair, teeth and nails
7	9	+	F	3 y 6 m	N	Sparse scalp hair, high anterior hairline, and sparse eyebrows.	Dry skin	D	O	+	Everted lower lip, deep labiomental sulcus, asymmetry of the lower lip, and bifid tip of the tongue.		Homozygous/ c.1788-1790delAGA p.(Glu596del)	Hair, teeth and nails
8	10	+	F	12 y 8 m	N	Sparse scalp hair * and high anterior hairline.	N	N	O	+	Thick lips, everted lips, narrow philtrum, narrow mandibular arch, lower pseudolabial cleft, retained deciduous teeth, and delayed eruption.		Homozygous/ c.1788-1790delAGA p.(Glu596del)	Hair and teeth

Abbreviations: Fa: family, P: patient, +: present, -: absent, F: female, M: male, N: normal, N/A: not applicable, D: dysplastic, and O: oligodontia. Variants’ nomenclature is based on *TSPEAR* (NM_144991.3, NP_659428.2) sequences. * Hypotrichosis was more prominent on the anterior part of the scalp.

**Table 3:** Population data, in silico variant effect prediction tools and ACMG classification of *TSPEAR* variants.

*TSPEAR* Variant Name NM_144991.3 NP_659428.2	Minor Allele Frequencies (MAF)	Polyphen-2	MutationTaster2	PROVEAN	SIFT	Mutation Assessor	CADD	GERP	ClinVar Clinical Significance	ACMG Classification/Evidence
c.44delC p.(Gly17Alafs*34	N/A	N/A	Disease-causing (Probability = 1)	N/A	N/A	N/A	N/A	1.91	N/A	Pathogenic (PVS1, PM2, PP3)
c.668C>T p.(Ser223Leu)	gnomAD:0.0007259 1000G:0.0008 dbSNP:0.000982 (rs149481227)	Possibly damaging (0.521)	Disease-causing (Probability = 0.99, score = 145)	Deleterious (−2.962)	Deleterious (0.02)	Medium (0.828)	25.9	5.11	Conflicting interpretations of pathogenicity	Likely pathogenic (PM1, PM2, PP3, PP4)
c.1788-1790delAGA p.(Glu596del)	gnomAD:0.0001204 1000G: N/A dbSNP: 0.00026 (rs782084367)	N/A	Disease-causing (Probability = 0.99)	N/A	N/A	N/A	N/A	3.44	N/A	Pathogenic (PS4, PM1, PM2, PM3, PP1)
c.1423G>C p.(Gly475Arg)	gnomAD:0.00003183 1000G: N/A dbSNP: 0.00005 (rs782056388)	Probably damaging (0.994)	Disease-causing (Probability = 0.99, score = 56)	Deleterious (−6.024)	Deleterious (0.01)	Medium (0.828)	24.5	5.18	Uncertain significance	Likely pathogenic (PM1, PM2, PM3, PP1, PP3)

Note: Polyphen-2 score is the probability that a substitution is damaging. MutationTaster2 provides the probability of the prediction, and a score in case of amino acid substitutions according to an amino acid substitution matrix. PROVEAN score ≤ −2.5 is predicted to be damaging. SIFT scores < 0.05 are considered deleterious or not tolerated. Mutation Assessor ranks the functional impact of missense variants as neutral, low, medium, and high, with scores from 0 to 1, high impact, i.e., deleterious variants are close to 1. CADD score ≥ 20 predicts the missense variant is among the top 1% of the most deleterious substitutions of the human genome. The GERP score values are positive for conserved positions/constrained loci; in the case of multiple base deletions, the highest score among the deleted bases is displayed. ACMG classification is assigned according to levels of evidence (reference in text). N/A: not available. Abbreviations of the in silico tools’ names are in text.

**Table 4:** Comparison between clinical phenotypes and dysmorphic facial features of *TSPEAR* cohorts of different ethnicities.

Reported Ethnicity	North African	Middle Eastern	European	Others *	Total	*p*-Value
Number of patients	10/38 (26.3%)	12/38 (31.6%)	9/38 (23.7%)	7/38 (18.4%)	38/38 (100%)	-
TSPEAR-associated phenotypes **						
ED	10/10 (100%)	4/12 (33.3%)	6/9 (66.7%)	2/7 (28.6%)	22/38 (57.9%)	0.0046
TA without other ectodermal features	0/10 (0%)	3/12 (25%)	3/9 (33.3%)	2/7 (28.6%)	8/38 (21.1%)	0.2799
SNHL	0/10 (0%)	5/12 (41.7%)	0/9 (0%)	2/7 (28.6%)	7/38 (18.4%)	0.0282
SNHL & ED	0/10 (0%)	0/12 (0%)	0/9 (0%)	1/7 (14.3%)	1/38 (2.6)	0.2080
**Dysmorphic facial features**						
Dysmorphic facial features	10/10 (100%)	5/12 (41.7%)	0/9 (0%)	2/7 (28.6%)	17/38 (44.7%)	0.0001
Detailed dysmorphic facial features	Ten ED patients of Egyptian origin featured: broad forehead short philtrum prominent and broad nasal root broad nose low set ears thick and everted lips and hypotrichosis was more prominent on the anterior of the scalp in six patients.	Five reported cases: 1- Three ED cases of Palestinian origin featured: long oval face down slanting of palpebral fissures low insertion of columella square chin thick lips and hypotrichosis was more prominent on the anterior of the scalp [27]. 2- One ED case of Saudi origin reported typical ED facial features including flat nasal bridge everted lips [29]. 3- One isolated TA case of Turkish origin had microcephaly, and reported faces were: narrow forehead high arched palate low set ears abnormal antitragus and increased hair growth on the forehead [28].	N/A	Two reported cases: 1- One ED case of Ashkenazi Jewish ancestry showed: long oval face down slanting of palpebral fissures low insertion of columella square chin thick lips and hypotrichosis was more prominent on the anterior of the scalp [27]. 2- The other case is African American who had TA, and featured: hypertelorism, depressed nasal bridge small and cupped ears [24].		
Ectodermal elements involvement in ED phenotype ***						
Teeth	10/10 (100%)	4/4 (100%)	3/6 (50%)	2/3 (66.7%)	19/23 (82.6%)	0.1737
Hair	9/10 (90%)	4/4 (100%)	3/6 (50%)	2/3 (66.7%)	18/23 (78.3%)	0.0477
Sweat glands	0/10 (0%)	4/4 (100%)	4/6 (66.7%)	1/3 (33.3%)	9/23 (39.1%)	0.0022
Nails	4/10 (40%)	0/4 (0%)	4/6 (66.7%)	2/3 (66.7%)	10/23 (43.5%)	0.1649
**References**	Current study	[24,25,27,28,29]	[24]	[24,27,55,56]		

Abbreviations: N/A: not available. * Others includes reports of African American, Ashkenazi Jewish, Asian, Korean, Caucasian or unspecified ethnicities. ** *TSPEAR*-associated phenotypes refers to the classification of patients as ED (two or more of the four classical ectodermal elements are affected including teeth) or TA (missing teeth with no other ectodermal elements involved) or SNHL (sensorineural hearing loss with no ED features) or SNHL & ED (sensorineural hearing loss accompanied by ED features). *** Ectodermal elements involvement in ED phenotype refers to the ectodermal element/s affected in the subset of patients classified as ED per each ethnicity.

## Data Availability

The data presented in this study are available on request from the corresponding author. Whole exome sequencing data are not publicly available due to ethical considerations of patients’ confidentiality.

## Supplementary Materials

The following supporting information can be downloaded at: https://www.mdpi.com/article/10.3390/genes13061056/s1, Figures S1–S9: Panoramic radiographs of P1–P8 & P10.
