# Supplementary material for: Confirmation of a Phenotypic Entity for TSPEAR Variants in Egyptian Ectodermal Dysplasia Patients and Role of Ethnicity
Source: Genes (Basel). 2022 Jun 13;13(6):1056. doi: 10.3390/genes13061056 (PMC9222913; doi:10.3390/genes13061056)
Supplement: Supplementary file 1 [file genes-13-01056-s001.zip › genes-1746524-supplementary.pdf]

Supplementary Figures

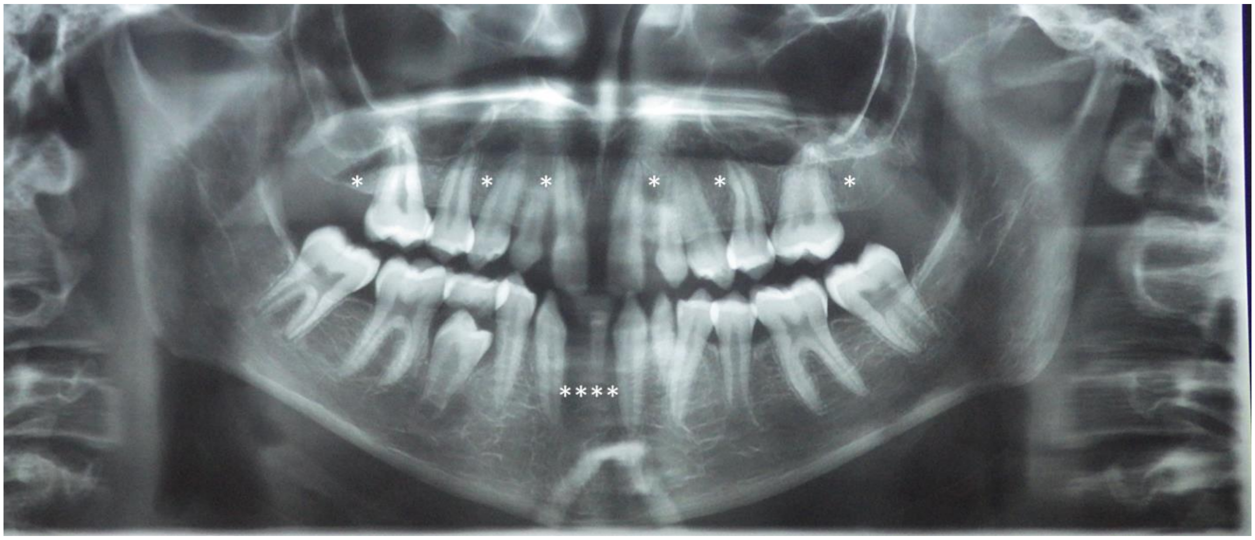

Supplementary Figure S1: Panoramic radiograph of P1 at the age of 16 years showing missing permanent upper lateral incisors, upper first premolars, upper second molars and all lower incisors, and retained deciduous upper canines and lower left central incisor. Delayed eruption of lower right second premolar and delayed dental development indicated by open apices of lower left second premolar and lower second molars.

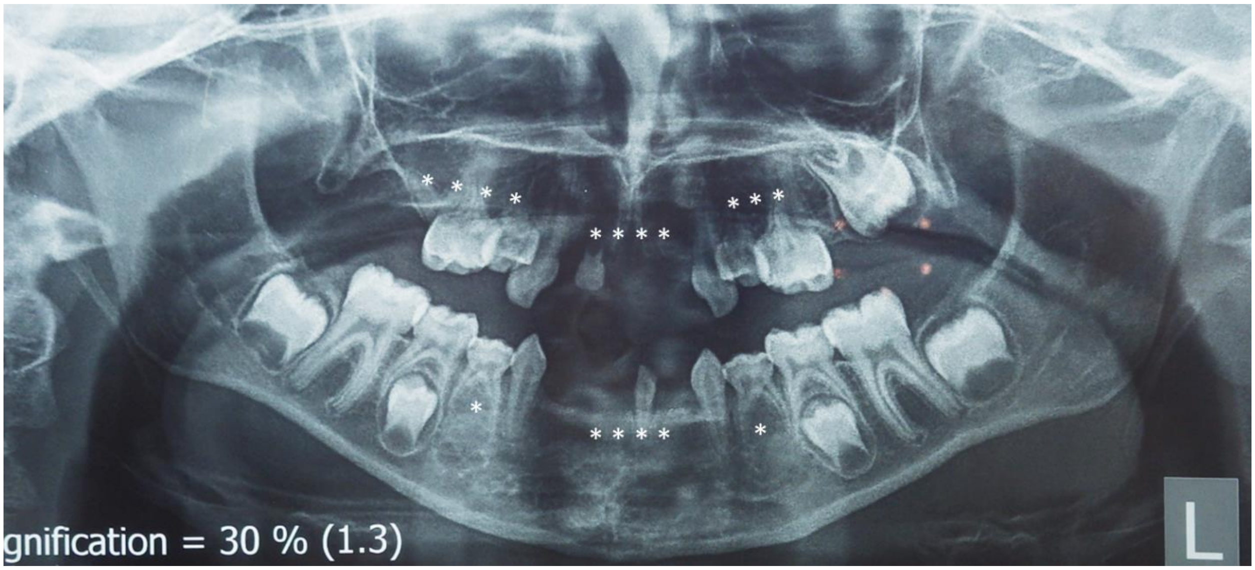

Supplementary Figure S2: Panoramic radiograph of P2 at the age of 8 years 10 months showing

missing all permanent upper and lower incisors, upper and lower first premolars, upper second premolars, first and second molars.

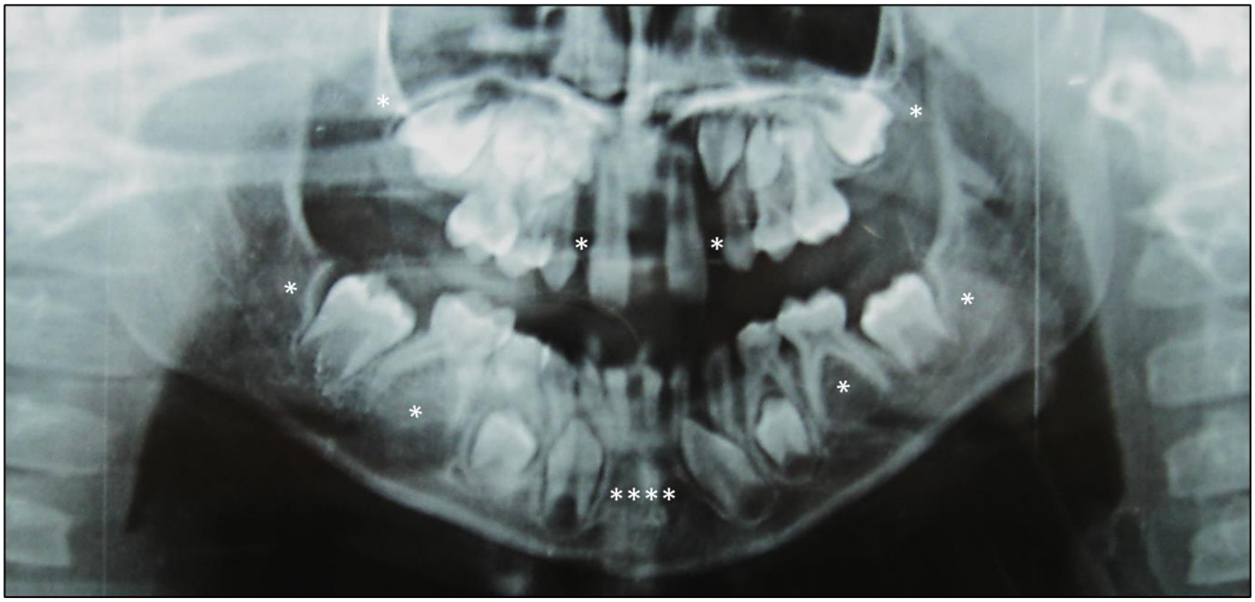

Supplementary Figure S3: Panoramic radiograph of P3 at the age of 9 years 5 months showing missing permanent upper lateral incisors and second molars, all lower incisors, lower second premolars and second molars. Delayed eruption of upper first molars and delayed dental development of lower first molars.

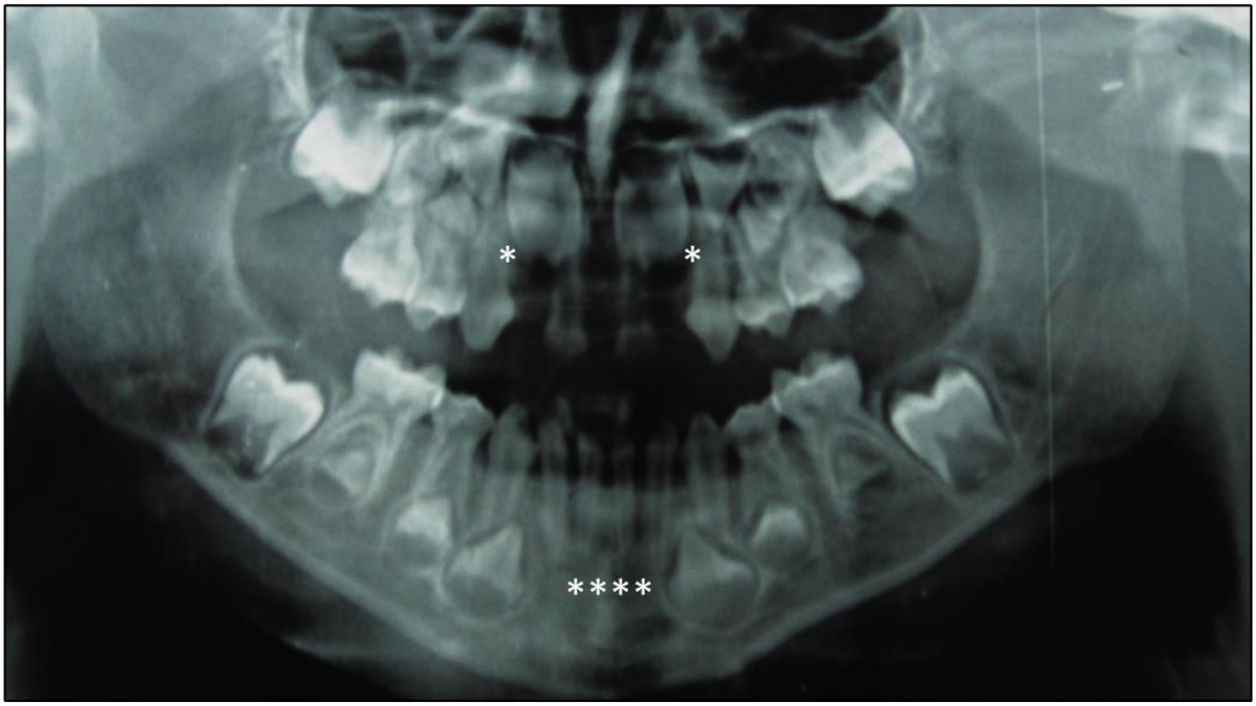

Supplementary Figure S4: Panoramic radiograph of P4 at the age of 6 years showing missing permanent upper lateral incisors and all lower incisors.

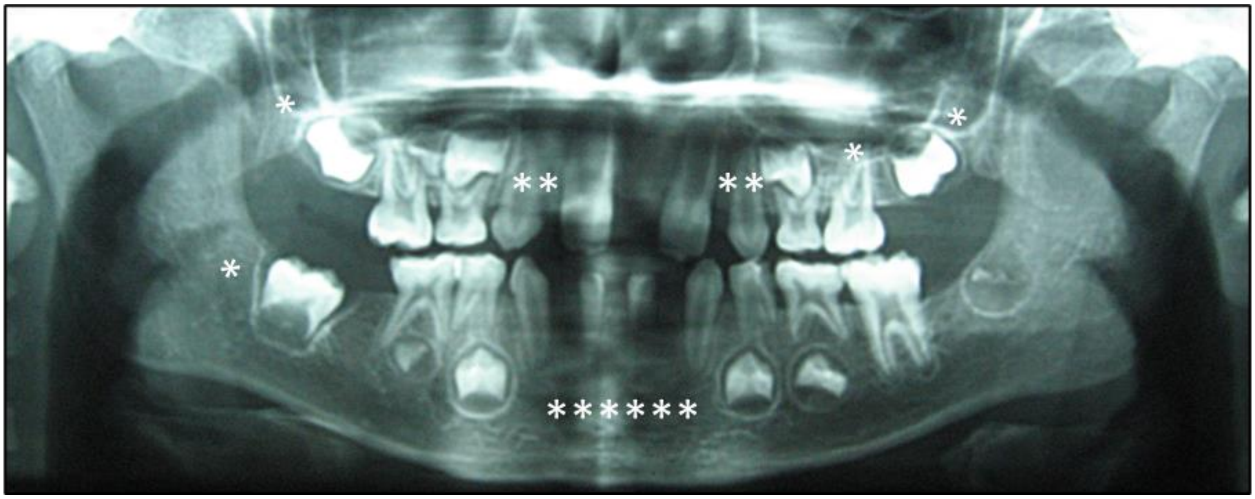

Supplementary Figure S5: Panoramic radiograph of P5 at the age of 7 years showing missing permanent upper lateral incisors, canines, left second molars and second molars and lower anterior teeth andl right second molar.

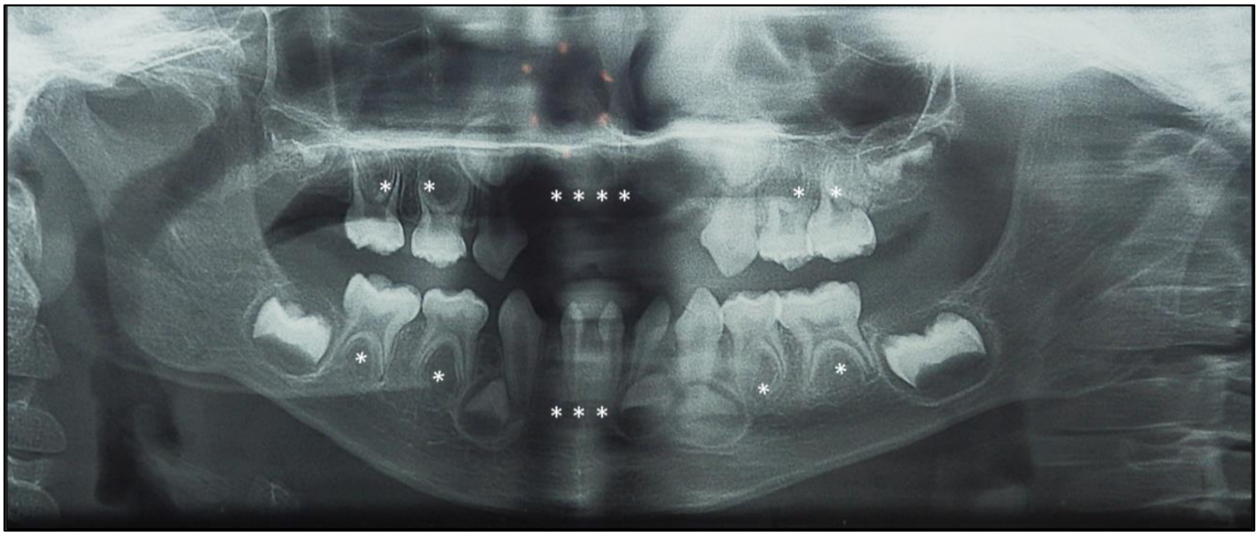

Supplementary Figure S6: Panoramic radiograph of P6 at the age of 4 years showing missing all permanent upper incisors, lower central incisors, lower right lateral incisor and all premolars.

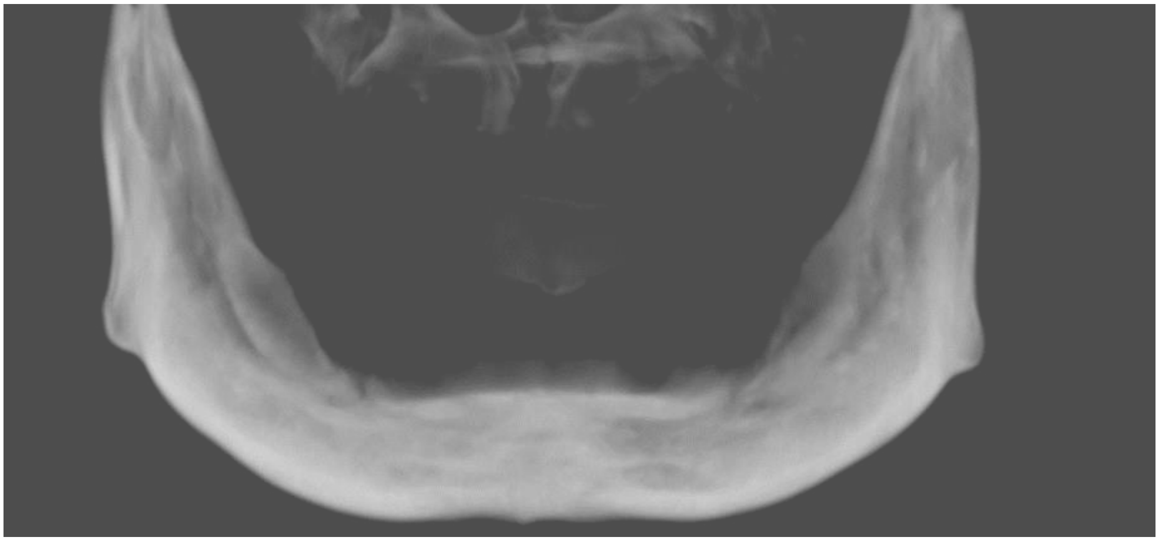

Supplementary Figure S7: Cone beam CT of P7 showing completely edentulous maxilla and mandible with severely atrophic maxilla. (N.B: Cone beam CT was done for P7 because it was needed prior to surgical intervention for bone grafting in the maxilla and panoramic radiograph would not give sufficient details for this surgery).

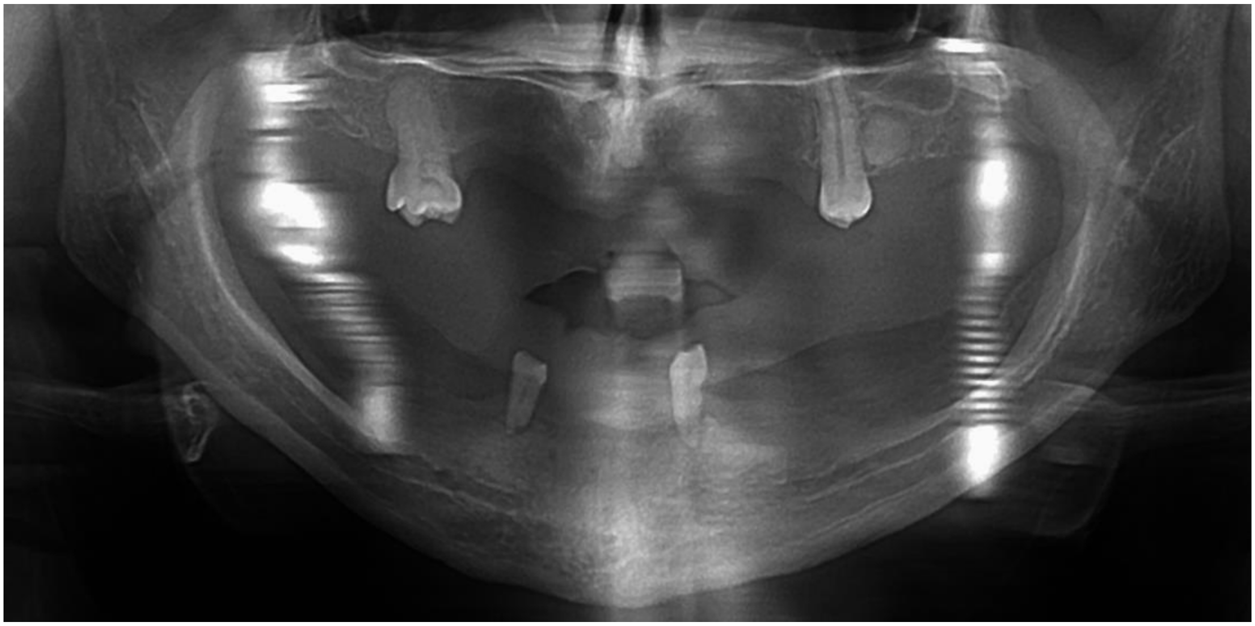

Supplementary Figure S8: Panoramic radiograph of P8 showing missing all permanent teeth except upper first molars and lower first premolars.

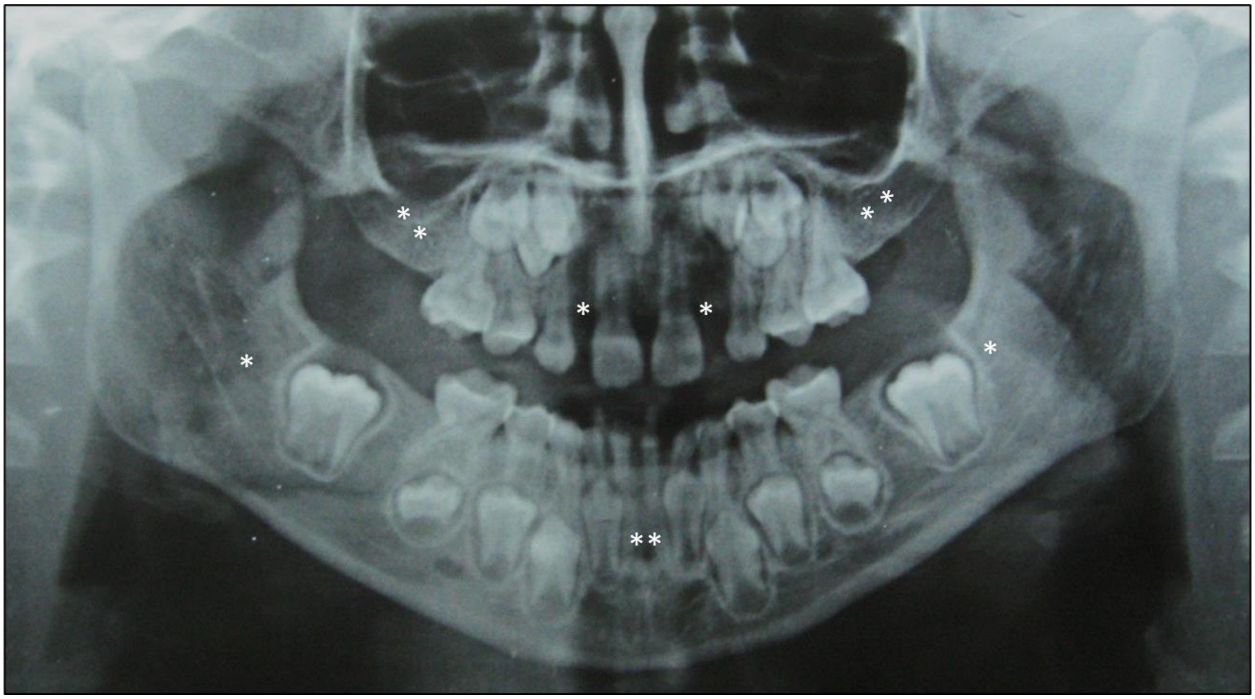

Supplementary Figure S9: Panoramic radiograph of P10 at the age of 8 years showing missing permanent upper lateral incisors, upper first and second molars, lower central incisors and lower second molars.
